# Bacterial Infections and Their Antibiotic Resistance Pattern in Ethiopia: A Systematic Review

**DOI:** 10.1155/2019/4380309

**Published:** 2019-08-05

**Authors:** Alemayehu Reta, Abebaw Bitew Kifilie, Abeba Mengist

**Affiliations:** Debre Markos University, College of Health Sciences, Department of Medical Laboratory Science, Ethiopia

## Abstract

**Background:**

Antibiotic resistance is a global challenge in the public health sector and also a major challenge in Ethiopia. It is truly difficult to report bacterial antibiotic resistance pattern in Ethiopia due to the absence of a review which is done comprehensively. The aim of this systematic review was to provide an overview of the works of literature on the antibiotic resistance pattern of the specific bacterial isolates that can be obtained from different clinical samples in the context of Ethiopia.

**Materials and Methods:**

A web-based search using PubMed, Google Scholar, Hinari, Sci Hub, Scopus and the Directory of Open Access Journals was conducted from April to May 2018 for published studies without restriction in the year of publication. Works of literature potentially relevant to the study were identified by Boolean search technique using various keywords: Bacterial infection, antimicrobial resistance, antibiotic resistance, drug resistance, drug susceptibility, anti-bacterial resistance, Ethiopia. Study that perform susceptibility test from animal or healthy source using <10 isolates and methods other than prospective cross-sectional were excluded.

**Results:**

The database search delivered a total of 3459 studies. After amendment for duplicates and inclusion and exclusion criteria, 39 articles were found suitable for the systematic review. All studies were prospective cross-sectional in nature. The review encompasses 12 gram-positive and 15 gram-negative bacteria with their resistance pattern for around 12 antibiotics. It covers most of the regions which are found in Ethiopia. The resistance pattern of the isolates ranged from 0% up to 100%. The overall resistance of* M. tuberculosis *for antituberculosis drugs ranges from 0% up to 32.6%. The percentage of resistance increases among previously treated tuberculosis cases.* Neisseria gonorrhea,* S. typhimurium, S. Virchow, Group A Streptococci (GAS), and Group B Streptococci (GBS) were highly susceptible for most of the tested antibiotics. Methicillin-Resistant* Staphylococcus aureus* was highly resistant to most of the antibiotics with a slightly increased susceptibility to gentamycin.

**Conclusions:**

Total bacterial isolates obtained from a different source of sample and geographic areas were 28, including* M. tuberculosis*. Majority of the bacterial isolates were resistant to commonly used antibiotics. A continuous monitoring and studies on the multidrug-resistant bacterial isolates are important measures.

## 1. Introduction

Human beings have been living unfriendly with a lot of microorganisms that can be a potential cause of infections and diseases. In the case of bacterial infections, due to the introduction of Penicillin for treatment in the early 1940s, there was an improvement [1]. Majority of naturally derived antibiotics are produced from Actinomycetes [2, 3]. In this day, even though the struggle to defeat bacterial pathogens continues, bacteria are evolving ever more clever by manifesting different forms of resistance [4].

The current antimicrobial profile studies have been proved that, bacteria that can cause nosocomial as well as community acquired infections become pan resistant for different groups of antibiotics. Hence, this situation becomes a clinical threat to the human beings [5–13]. Most of the bacterial antibiotic resistance mechanisms are acquired by altering of target genes or acquisition of plasmid encoding resistance genes. These encoded genes may lead to the production of lytic enzymes, change of membrane permeability, efflux action, and hiding from the action of antibiotics [14].

Centers for Disease Control and Prevention (CDC) stated that antibiotic resistance is responsible for around 2 million infections, more than twenty thousand deaths and, costs $55 billion each year in the United States [15]. The national pharmaceutical sales data on global antibiotic consumption (2000-2010) reveals that total antibiotic consumption grew by more than 30%. The greatest increase in antibiotics use was recorded in Low and Middle Income Countries (LMIC) [16].

As long as Ethiopia is one of the LMICs, antibiotic resistance is a major challenge. Yet, there is no antibiotic stewardship that helps to establish surveillance system for tracking current antibiotic use and its resistance in Ethiopia. Therefore, it is truly difficult to report bacterial antibiotic resistance pattern in Ethiopia. So, the aim of this systematic review emphasizes on the antibiotic resistance pattern of the specific bacterial isolates that can be obtained from different clinical samples in the context of Ethiopia.

## 2. Materials and Methods

### 2.1. Eligibility Criteria

All available studies and data were incorporated based on the following predefined eligibility criteria:

1. should be published and written in English,
2. had to describe the microbial isolation, identification, and antimicrobial sensitivity test methods according to the criteria of the Clinical Laboratory Standards Institute (CLSI) and defined antimicrobial resistance range according to CLSI manual,
3. studies which used human infection sample (isolated from a diseased individual),
4. had to report the number of tested isolates (>10) and the number of isolates resistant or sensitive,
5. should be a prospective cross-sectional study.

### 2.2. Study Selection Procedure and Search

**Figure 1:**
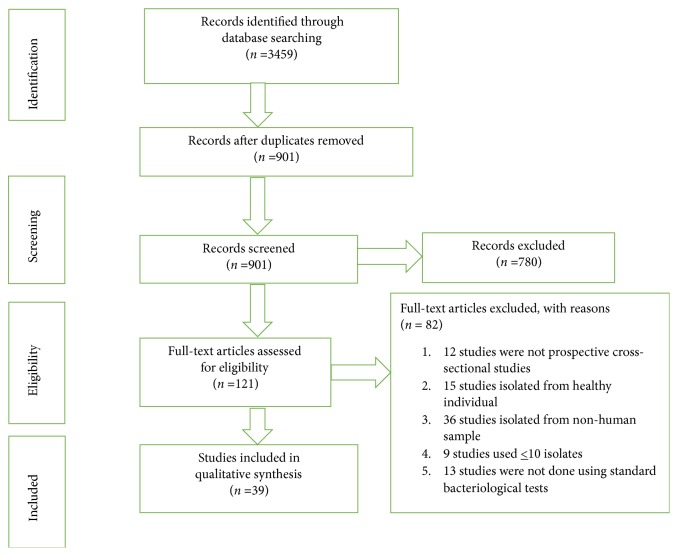
A flow diagram of the selection of eligible studies. The flow diagram shows the procedure of selecting an eligible study to undergo the systematic review. To perform this we start by identifying 3459 studies using a web-based search and goes to a screening of 901 studies after removing duplicates. Using eligibility criteria only 121 studies went to eligibility testing. Finally, 39 studies were included.

The search and selection of eligible studies were shown in Figure 1. To make it more elaborative; a web-based search using PubMed, Google Scholar, Hinari, Sci Hub, Scopus, and the Directory of Open Access Journals (DOAJ) was conducted from April to May 2018 and pieces of literature potentially relevant to the study were identified. The search was performed using various keywords: Bacterial infection, antimicrobial resistance, antibiotic resistance, drug resistance, drug susceptibility, anti-bacterial resistance, Ethiopia. These key terms were used in various combinations using Boolean search technique. The search was not limited to the year of publication.

Relevant search results from the above site were individually downloaded and the reference lists of the identified studies were used to scrutinize to identify extra articles.

### 2.3. Data Extraction

Essential data were extracted from eligible studies using Excel spreadsheet format prepared for this purpose and any discrepancies were resolved by the author. The data extracted from eligible studies include the name of regions, study area/city, the name of the author(s), year of the study, study design, types of specimens, numbers of patients/study participants, number of resistant isolates, resistance pattern of the isolates, and references.

## 3. Results

### 3.1. Literature Search Results

The search from PubMed, Google Scholar, Hinari, Sci Hub, Scopus, and DOAJ delivered a total of 3459 studies. After amendment for duplicates, 901 endured. Of these, 780 studies were castoff, since, after a review of their titles and abstracts, they did not meet the criteria. The full texts of the remaining 121 studies were reviewed in detail. Of these, 85 studies were discarded after the full text had been reviewed for appropriate study method, sample source, number of isolates, and standard bacteriological test. Finally, 39 studies were included in the review (Figure 1).

### 3.2. Antibiotic Resistance for Gram-Positive Bacteria

**Figure 2:**
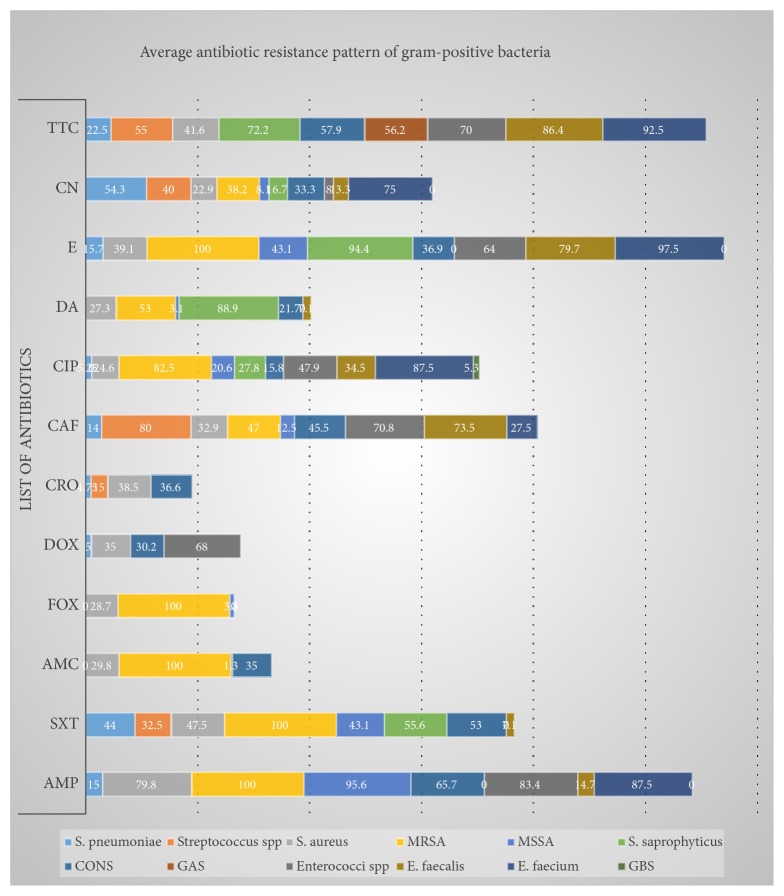
Average resistance pattern of gram-positive bacteria for different antibiotics.

The review tries to encompass 12 gram-positive bacteria and their resistance pattern for around 12 antibiotics. It covers most of the regions which are found in Ethiopia. The resistance pattern of the isolates ranged from 0% up to 100%. GAS and GBS were highly susceptible for most of the tested antibiotics, but they have a relatively increased resistance to tetracycline. In contrast with these, MRSA was highly resistant for most of the antibiotics with a slightly increased susceptibility to gentamycin. The rest bacterial isolates have a different resistance pattern for different antibiotics and also a variable pattern from sample to sample (Table 1). The average resistance pattern of gram-positive bacteria shows a cumulative antibiotic resistance pattern of bacterial isolates from different clinical cases, geographic location, and source of sample for a similar antibiotic. Still, the average resistance range of the gram-positive bacteria is similar with the detailed resistance pattern (Figure 2).

### 3.3. Antibiotic Resistance for Gram-Negative Bacteria

**Figure 3:**
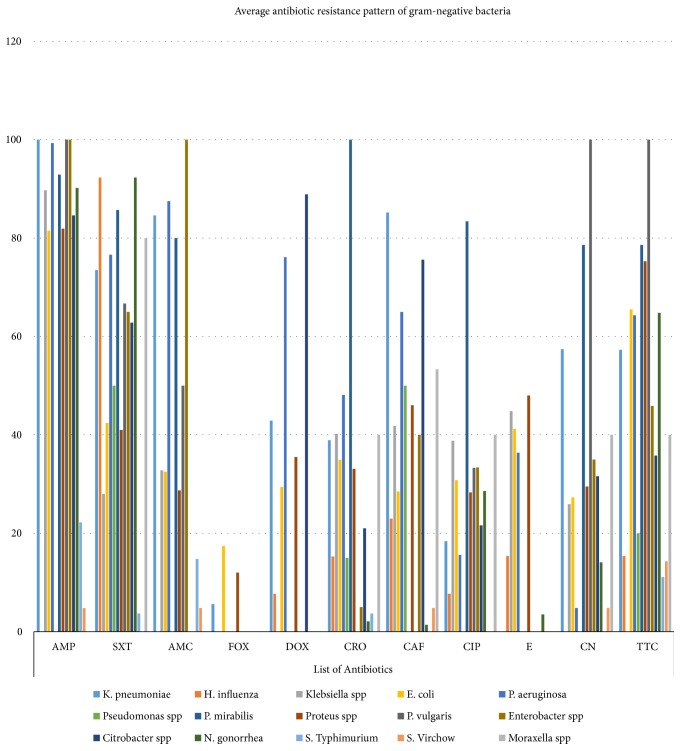
Average resistance pattern of gram-negative bacteria for different antibiotics.

Fifteen gram-negative bacteria were recovered from various specimens. Like the gram-positive bacteria, the resistance pattern ranges from 0% up to 100%. Almost all bacterial isolates were highly resistant for ampicillin. Relatively, isolates obtained from conjunctival swab were highly susceptible to different antibiotics.* Neisseria gonorrhea, S. typhimurium*, and S. Virchow were susceptible for many antibiotics.* Moraxella* spp. and S. Virchow had a similar resistance pattern for different antibiotics, even if the number of isolates varies between the two (Table 2). The average resistance pattern of gram-negative bacteria shows a cumulative antibiotic resistance pattern of bacterial isolates from different clinical cases, geographic location, and source of sample for a similar antibiotic. Still, the average resistance range of the gram-negative bacteria is similar with the detailed resistance pattern (Figure 3).

### 3.4. Drug Resistance of* M. tuberculosis*

The overall resistance of* M. tuberculosis *for antituberculosis drugs ranges from 0% up to 32.6%. The percentage of resistance increases among previously treated tuberculosis cases. New pulmonary and extra-pulmonary cases relatively had decreased resistance for antituberculosis drugs (Table 3).

## 4. Discussion

Our results indicate that the antibiotic resistance pattern of both gram-negative and gram-positive bacteria varied across the studies reviewed, ranging from 0% to 100%. This variation was found depending on the type of isolate, the source of the sample, type of infection, type of antibiotics, and geographical difference used in each study.

Even though it is difficult to discuss average resistance pattern of gram positive and gram negative bacteria with a single study for various antibiotics, a study done in Nigeria revealed that gram-negative and gram-positive bacteria had a resistance pattern of 19.8%-92.3% and 10%-87%, respectively [17]. And also a study in Gondar, Northwest Ethiopia, showed 20%–100% and 23.5%–58.8% for gram-negative and gram-positive bacteria, respectively [18]. If we look at the overall resistance pattern of the above studies, it ranges from 10% to 100%. This was relatively comparable to the current review result.

A systematic review done on antimicrobial resistance in Sub-Saharan Africa (1990–2013) has been reported that Gram-positive pathogens show high prevalence of resistance to chloramphenicol, trimethoprim/sulfamethoxazole, and tetracycline and gram negative bacteria specifically the enterobacteriaceae groups show resistance to chloramphenicol, isolated from patients with a febrile illness, ranged between 31.0% and 94.2%, whereas resistance to third-generation cephalosporins ranged between 0.0% and 46.5% [13].

The current review revealed that majority of the bacterial isolates are resistant to commonly used antibiotics in Ethiopia [19–54]. The possible reason might be related to scientific justifications like the following: numerous antibacterial agents, effective previously, are no longer used today because of the rise of resistance genes in the bacterial genome [55]. The emergence of resistance genes can be through natural selection in the environment over a long period of time or by a spontaneous mutation in the bacterial DNA. The resistant pattern has been reported by almost all antibiotics that have been developed [19].

## 5. Limitations of the Study

Most of the studies done in Ethiopia do not document the antibiogram; due to this we were unable to review and extract data related to multidrug resistance.

## 6. Conclusions

The main result of this study is to obtain an internationally valid reference to know the antibiotic resistance pattern in Ethiopia. The review encompasses 12 gram-positive bacteria and their resistance pattern for around 12 antibiotics. It covers most of the regions which are found in Ethiopia. The resistance pattern of the isolates ranged from 0% up to 100%. Fifteen gram-negative bacteria were recovered from various specimens. Like the gram-positive bacteria, the resistance pattern ranges from 0% up to 100%. Almost all bacterial isolates were highly resistant for ampicillin. Relatively, isolates obtained from conjunctival swab were highly susceptible to different antibiotics.* Neisseria gonorrhea*,* S. typhimurium*, and S. Virchow were susceptible for many antibiotics. The overall resistance of* M. tuberculosis* for antituberculosis drugs ranges from 0% up to 32.6%. Given the limitations of the current study, these findings should be interpreted carefully but warrant further evaluation in consequent studies.

## 7. Recommendations

Our study provides evidence that majority of the bacterial isolates were resistant to commonly used antibiotics. Antibiotic resistance should be a substantial concern for Ethiopia as well as for all countries around the globe. So to alleviate such problems and to advance the effectiveness of antibiotics in Ethiopia, the government of Ethiopia as well as the international community should do the following:

- Prepare the guidelines for proper use of antibiotics in the health institutions.
- Establish antimicrobial resistance stewardship.
- Increase proper immunization coverage that may reduce the use of antibiotics.
- Implement one health policy (reduce antimicrobial use for agricultural practice and animals).
- Create community awareness on rational use of drugs.
- Make strong policy for antibiotic dispensing by drug venders.
- Control Hospital and community acquired infections.
- Ensure political commitment to meet the threat of antibiotic resistance.

## Figures and Tables

**Table 1:** Antibiotic resistance for gram-positive bacteria.

Microorganisms	Type of sample	Study area	No of isolates	Antibiotics and resistance (%)												Reference
				AMP	SXT	AMC	FOX	DOX	CRO	CAF	CIP	DA	E	CN	TTC	
*S. pneumoniae*	Nasal swab	GUH	96	10	22	-	0	-	0	14	2	-	32	-	32	[20]
	Conjunctival swabs	West Gojjam	25	-	44	-			4	32	4			8	8	[21]
	Ocular swab	JUSH	20		65			5	15	0	5		10	85	20	[22]
	Ocular swab	HUH	20	20	45	0			0	10	10		5	70	30	[23]

Streptococcus spp	Conjunctival swabs	West Gojjam	40		32.5				15	80	0			40	55	[21]

*S. aureus*	Urine	Bahir Dar	10	100	87.5						33.3			50	60	[24]
	Blood	Mekelle	54		66.7	13		53.7	57.4		38.9		44.4	33.3		[25]
	Nasal swab	Adigrat, Wukro	29	48.3	51.7		48.3			17.2	37.9	17.2	62.1	37.9	55.2	[26]
	Ocular swab	JUSH	42		21.4			19.1	23.8	57.1	4.8		35.7	4.8	21.4	[22]
	Urine	GUH	28	100	100				100	50	100			50	0	[27]
	Wound	JUSH	73	94.5	60.3		28.8	82.2	20.5	69.9	13.7	85	79.4	16.5		[28]
	Ear discharge	HUCSH				48.5								18.2		[29]
	Wound	JUSH	47	95.7	6			27.6	14.9	14.9	4		14.9	4	51	[30]
	Pus*∗*	GGH	15	80	26.7	26.7	20	0	40	20	20	20	26.7	0	13.3	[31]
	Blood	GUH	17	47	58.3				35.3	23.5	29.4		58.8	29.4	35	[32]
	Eye discharge	GUH	13	76.9	69.2	53.8			23.1	30.8	15.4		46.2	46.2	61.6	[33]
	Wound	Mekelle	40	90		50			90				22.5	22.2	90	[34]
	Ocular swab	HUH	30	70	23.3	10			6.7	6.7	6.7		16.7	6.7	60	[23]
	Ocular specimen	QOH	40		42.5			27.5		27.5	12.5	22.5	20	12.5	45	[35]
	Ocular specimen	GUH	96	92.7	7.3	17.7			20.8	11.5	7.3	7.3	28.1	28.1	27.1	[36]
	Conjunctival swabs	West Gojjam	120	66	38				30	80	13			16.6	21	[21]
	Specimen*∗*	YHMC	194	96.4	53.1	18.5	17.5			18.6	31.4	11.9	53.1	13.4		[37]

MRSA	Specimen*∗*	YHMC	34	100	100	100	100			47	82.5	53	100	38.2		[37]

MSSA	Specimen*∗*	YHMC	160	95.6	43.1	1.3	3.8			12.5	20.6	3.1	43.1	8.1		[37]

*S. saprophyticus*	Urine	AAML	18		55.6						27.8	88.9	94.4	16.7	72.2	[38]

CONS	Blood	GUH	30	40	53.3				36.6	40	23.3		30	30	73.3	[32]
	Ocular swab	JUSH	15		46.6			6.7	26.7	73.4	0		26.7	53.3	33.3	[22]
	Eye discharge	GUH	17	76.5	70.6	64.7			23.5	52.9	23.5		64.7	58.8	64.7	[33]
	Blood	Mekelle	44		81.8	11.4		61.4	50		25		40.9	18.1		[25]
	Ocular specimen	GUH	64	89.1	23.4	17.2			17.2	15.6	10.9	4.7	42.2	26.6	32.8	[36]
	Wound	Mekelle	18	77.8		77.8			72.3				50	50	77.8	[34]
	Ocular swab	HUH	26	30.8	38.5	3.8			26.7	26.9	18.2		11.5	23.1	65.4	[23]
	Conjunctival swabs	West Gojjam	110	80	41.8				40	81						[21]
	Ocular specimen	QOH	31		67.7			22.6		29	9.7	38.7	29	6.5	58.1	[35]

GAS	Throat swab	Addis Ababa	52	0	0		0					0	0		34.6	[39]
		Dire Dawa	16	0	0		0					0	0		75	[39]
		Gondar	22	0	0		0					0	0		59.1	[39]

Enterococci spp	Conjunctival swabs	West Gojjam	25	100							25			8	72	[21]
	Urine, wound, blood	GUH	24	66.7				68		70.8	70.8		64		68	[40]

*E. faecalis*	Urine	AAML	14		7.1					73.5	7.1	7.1	85.8	0	78.6	[38]
	Stool, rectal swab	Jimma	34	14.7						73.5	61.8		73.5	26.5	94.1	[41]

*E. faecium*	Stool, rectal swab	Jimma	40	87.5						27.5	87.5		97.5	75	92.5	[41]

GBS	Vaginal swab	Mekelle	19	0							5.3		0	0		[42]

Specimen*∗*: Nasal swab, pus from wound, ear discharge, blood, throat swab, eye swab, vaginal discharge, urethral discharge, urine, stool, sputum, CSF, and body fluids; Pus*∗*: collected from leprosy ulcer; AMP: Ampicillin; SXT: Trimethoprim-Sulfamethoxazole; AMC: Amoxicillin-Clavulanic acid; Dox: Doxycycline; CRO: Ceftriaxone; CAF: Chloramphenicol; CIP: Ciprofloxacin; DA: Clindamycin; E: Erythromycin; CN: Gentamicin; TTC: Tetracycline; FOX: Cefoxitin; GUH: Gonder University Hospital; JUSH: Jimma University Specialized Hospital; CONS: Coagulase-negative Staphylococcus; HUH: Hawassa University Hospital; GAS: Group A Streptococcus; GBS: Group B Streptococcus YHMC: Yekatit 12 Hospital Medical College; QOH: Quiha Ophthalmic Hospital; AAML: Arsho Advanced Medical laboratory; HUCSH: Hawassa University Comprehensive Specialized Hospital MRSA: Methicillin-Resistant *Staphylococcus aureus*; MSSA: Methicillin-Sensitive *Staphylococcus aureus*

**Table 2:** Antibiotic resistance for gram-negative bacteria.

Microorganisms	Type of sample	Study area	No of isolates	Antibiotics and resistance (%)											Reference
				AMP	SXT	AMC	FOX	DOX	CRO	CAF	CIP	E	CN	TTC	
*K. pneumoniae*	sputum, urine, pus	Harar	57		65				40	70			61		[43]
	Urine	Bahir Dar	17	100	50	84.6					21		40	27.8	[24]
	Urine	AAML	18	100	66.7		5.6		44.4		16.7		22.2	44.4	[38]
	Urine	GUH	28	100	100				0	100	0		100	100	[27]
	Wound	JUSH	14	100	85.7			42.9	71	85.7	35.7		64	57	[30]

*H. influenza*	Ocular swab	JUSH			92.3			7.7	15.3	23	7.7	15.4	0	15.4	[22]

Klebsiella spp	Wound	Mekelle	29	89.7		65.5			86.2		37.9	44.8	27.8		[34]
	Conjunctival swab	West Gojjam	20		28				20	55	0		0	0	[21]
	Urine	AAHFH	14			0			14.3	28.6	78.6		50		[44]

*E. coli *	Urine	Bahir Dar	64	89.1	64.5	78.6					64.4		27.6	66.1	[24]
	Blood	Mekelle	16		6.7	6.7		40	60		6.7		13.3		[25]
	Urine	GUH	28	66.7	66.7				50	16.7	50		66.7	100	[27]
	Vaginal swab	FRH	15	73.3	60						20		26.7	73.3	[45]
	Wound	JUSH	30	76.7	20			23.3	66.7	13.3	3.3		0		[28]
	Wound	JUSH	29	100	55			44.8	62	65.5	34		51.7	79	[30]
	Urine	AAML	135	77.8	70.4	45.2	22.9		34.8		50.4		28.1	69.6	[38]
	Urine	GUH	19	100	26.3	36.8			0	0	0		5.3	52.6	[18]
	Urine	StHMC	53	79.2	22.6			71.7	45.3	30.2	54.7		22.6	83	[46]
	Conjunctival swab	West Gojjam	20		15				0	35	0		20	25	[21]
	Pus*∗*	GGH	17	70.6	58.8	5.9	11.8	29.4	5.9	35.3	29.4	41.2	11.8	41.2	[31]
	Urine	AAHFH	65			21.6			24.6	32.2	56.9		53.8		[44]

*P. aeruginosa*	Urine	Bahir Dar	8	100	71.4	75					0		0	40	[24]
	Wound	JUSH	74	97.3	87.9			83.3	9.5	74.3	5.4		10.8		[28]
	Ocular swab	JUSH	31		74.2			45.1	19.4	38.7	6.4		0	71	[22]
	Wound	JUSH	11	100	73			100	63.6	82	0		18	82	[30]
	Urine	JUSH	36								0		0		[47]
	Wound	Mekelle	11	100		100			100		81.8	36.4	0		[34]

*Pseudomonas spp *	Conjunctival swabs	West Gojjam	20		50				15	50	0		0	20	[21]

*P. mirabilis *	Urine	Bahir Dar	7	85.7	71.4	80					66.7		57.1	57.1	[24]
	Urine	GUH	28	100	100				100	0	100		100	100	[27]

*Proteus spp*	Wound	JUSH	23	91	39			43	65	30	17		26	74	[30]
	Wound	Mekelle	15	86.7		46.7			73.3		46.7		20		[34]
	Pus*∗*	GGH	25	68	56	20	12	28	8	36	32	48	32	96	[31]
	Conjunctival swabs	West Gojjam	25		28				0	76	0		8	56	[21]
	Urine	AAHFH	31			19.4			19.4	41.9	45.9		61.3		[44]

*P. vulgaris*	Urine	Bahir Dar	3	100	66.7	50					33.3		100	100	[24]

Enterobacter spp	Urine	Bahir Dar	3	100	100	100					66.7		50	66.7	[24]
	Conjunctival swabs	West Gojjam	20		30				5	40	0		20	25	[21]

Citrobacter spp	Urine	GUH	28	69.2	38.5				46.2	76.9	53.8		61.5	61.5	[27]
	Wound	JUSH	18	100	100			88.9	16.7	100	11.1		33.3		[28]
	Conjunctival swabs	West Gojjam	20		50				0	50	0		0	10	[21]

*Neisseria gonorrhea*	Urethral discharge	Gondar Health Center	142	80.3	92.3				4.2	1.4		3.5	14.1	29.6	[48]
	Urethral or endo-cervical swabs	Gambella hospital	21	100					0		28.6			100	[49]

S. Typhimurium	Stool	Addis Ababa	27	22.2	3.7	14.8	0		3.7	0	0		0	11.1	[50]

S. Virchow	Stool	Addis Ababa	21	4.8	0	4.8	0		0	4.8	0		4.8	14.3	[50]

Moraxella spp	Conjunctival swabs	West Gojjam	15		80				40	53.3	40		40	40	[21]

GUH: Gonder University Hospital; StHMC: St. Paul's Hospital Millennium Medical College; AAHFH: Addis Ababa Hamlin fistula hospital; FRH: Felegehiwot Referral Hospital; GGH: Gambo General Hospital Pus*∗* = collected from leprosy ulcer

**Table 3:** Resistance pattern of *M. tuberculosis* for anti-tuberculosis drugs.

Type of tuberculosis case	Type of sample	Study area	No of isolates	Antibiotics and resistance (%)					Reference
				H	R	S	E	P	
New case	Sputum	BLUH	103	8.7	1.9	7.8	0.9	-	[51]
Previously treated	Sputum	BLUH	18	5.6	5.6	5.6	5.6	-	[51]
New case	Sputum	JUSH	136	13.2	2.2	8.1	5.2	-	[52]
New case	Sputum	Amhara region	93	3.2	0	20.4	0	-	[53]
New case	Sputum	Amhara region	214	9.8	3.7	6.5	5.6	3.7	[54]
Previously treated	Sputum	Amhara region	46	32.6	15.2	26.1	15.2	8.7	[54]
EPTB	pleural, peritoneal and synovial fluids	Addis Ababa	58	8	21.6	5.4	2.7	-	[56]

H: isoniazid; R: rifampin; S: streptomycin; E: ethambutol; P: pyrazinamide; EPTB: Extra Pulmonary Tuberculosis; JUSH: Jimma University Specialized Hospital; BLUH: Black Lion University Hospital
